# 

**Published:** 1858-07

**Authors:** 


					﻿No. II.
JULY—1858.
Vol. XIV.
BUFFALO
MEDICAL JOURNAL
A X D
lllontljh) lleuiew
OF
MEDICAL AND SURGICAL SCIENCE,
AUSTIN FLINT, Jr., M. D.,
EDITOR.
BUFFALO, N. Y.
E. R. JEWETT, PUB El SHER.
Commercial Advertiser Buildings.
Price $2.50, payable in advance, or $3.00 if not paid till the end of the year.
				

